# Barriers to using HIV pre-exposure prophylaxis (PrEP) and sexual behaviour after stopping PrEP: a cross-sectional study in Germany

**DOI:** 10.1186/s12889-021-10174-4

**Published:** 2021-01-19

**Authors:** Uwe Koppe, Ulrich Marcus, Stefan Albrecht, Klaus Jansen, Heiko Jessen, Barbara Gunsenheimer-Bartmeyer, Viviane Bremer

**Affiliations:** 1 Department of Infectious Disease Epidemiology, Robert Koch Institute, Berlin, Germany; 2 Department of Epidemiology and Health Monitoring, Robert Koch Institute, Berlin, Germany; 3 Praxis Jessen2 und Kollegen, Berlin, Germany

**Keywords:** HIV pre-exposure prophylaxis, Men, Who have sex with men, Former use, Condom use, Side effects

## Abstract

**Background:**

Persistence of individuals at risk of HIV with Pre-Exposure Prophylaxis (PrEP) is critical for its impact on the HIV epidemic. We analysed factors associated with stopping PrEP, barriers that may deter people from continuing PrEP and investigated sexual behaviour after stopping PrEP.

**Methods:**

Current and former PrEP users in Germany were recruited to complete an anonymous online survey on PrEP use and sexual behaviour. Participants were recruited through dating apps, a PrEP community website, anonymous testing sites and peers. The results were analysed using descriptive methods and logistic regression.

**Results:**

We recruited 4848 current and 609 former PrEP users in two study waves (July–October 2018, April–June 2019). Former PrEP users were more likely 18–29 years old than current users (adjusted OR = 1.6, 95% confidence interval (CI) 1.1–2.3). Moreover, they were more often unhappy with their sex life, which was more pronounced in former daily PrEP users (aOR = 4.5, 95% CI 2.9–7.1) compared to former on-demand users (aOR = 1.8, 95% CI 1.1–2.9, p_interaction_ = 0.005). The most common reason for stopping PrEP was a reduced need for PrEP (49.1%). However, 31.4% of former users identified logistic reasons and 17.5% stopped due to side effects. Former PrEP users using PrEP < 3 months were more likely to stop PrEP due to concerns over long-term side effects (32.0% vs. 22.5%, *p* = 0.015) and not wanting to take a chemical substance (33.2% vs. 24.0%, *p* = 0.020) compared to former PrEP users who used PrEP for longer. After stopping PrEP, 18.7% of former PrEP users indicated inconsistent condom use while having ≥4 sex partners within the previous 6 months. Former PrEP users with many partners and inconsistent condom use more often indicated logistic reasons for stopping (46.5% vs. 27.9%, *p* < 0.001) than did other former PrEP users.

**Conclusions:**

To maximise persistence with PrEP we need to develop strategies for younger PrEP users, reduce logistic barriers to access PrEP, and to develop effective communication on side-effect management. Moreover, prevention strategies for people stopping PrEP are required, since some remain at high risk for HIV.

**Supplementary Information:**

The online version contains supplementary material available at 10.1186/s12889-021-10174-4.

## Introduction

HIV pre-exposure prophylaxis (PrEP) with Tenofovirdisoproxil/Emtricitabine (TDF/FTC) is a powerful tool for HIV prevention and is recommended by national and international guidelines [1–3]. Widespread roll-out was associated with a reduction in new HIV diagnoses in men who have sex with men (MSM) in Australia, Paris, London, and the United States [4–8]. In Germany, generic PrEP for 40–70€ self-pay per month became available in 2017. Statutory health insurances have reimbursed PrEP since September 2019, which is expected to increase the uptake of PrEP.

PrEP programmes need to ensure that people stay on PrEP for as long as they are at risk. Ensuring persistence with PrEP was associated with the biggest impact on HIV incidence [9], yet many users discontinue PrEP within the first year after initiation with some studies finding that less than half were retained [10–12].

The reasons for stopping PrEP are diverse. Some users change their sexual behaviour or only have risky sex during certain periods in their lives [13, 14]. Other people stop PrEP because of adverse drug effects [15]. Other reasons for stopping PrEP indicate access barriers, e.g. lack of health insurance cover, language barriers, self-payments, or frequent doctor visits [16–20]. Moreover, fears of side effects, experiencing stigma, and fears of insufficient protection by PrEP could also keep some from continuing their use of PrEP [16, 21, 22].

Here, we investigate factors associated with stopping PrEP and identify barriers that may be addressed in order to maximise PrEP persistence. Moreover, we investigate the sexual behaviour of former PrEP users to inform HIV prevention strategies.

## Methods

### Study design

The PrApp study is a cross-sectional study investigating PrEP use in Germany [23]. Current and former PrEP users were recruited on dating apps, a community website, in anonymous testing sites and through peers. After providing consent on the survey website, participants completed an anonymous online survey concerning PrEP use, sexual behaviour, reasons for stopping PrEP and side effects. The study was rolled out in two waves in 2018 and 2019.

### Outcomes and covariates

The outcomes of this study included factors associated with former PrEP use, reasons for stopping PrEP and self-reported side effects.

The survey questions are shown in Additional file 1: Appendix S1. Regarding demographic characteristics, age groups were defined as 18–29, 30–39 and 40–49 years. Participants aged 50+ years were grouped together because of sparse data. Gender was categorised into male, female, transgender/non-binary (identifying as transgender, non-binary or with gender identity discordant from sex assigned at birth), and intersex (identifying as intersex or assigned intersex at birth). Sex assigned at birth was only included in wave 2, so gender identities based on this variable could not be inferred for wave 1 participants. Country of origin was grouped into the binary variable ‘Germany’ and ‘Outside Germany’. The annual gross income was categorised into < 30,000€, 30,000–49,000€, and ≥ 50,000€. Satisfaction with sex life was grouped into a binary variable to reduce complexity in the dataset combining the answers very happy and happy to the category ‘Happy’ and the answers very unhappy and unhappy to the category ‘Unhappy’. Regarding variables for sexual behaviour, the numbers of anal/vaginal sex partners within the previous 6 months were categorised into 0–3, 4–10, and > 10 partners. Concerning condom use, the categories ‘Always’ and ‘Often’ were grouped together, as were the categories ‘About half the time’, ‘Sometimes’, and ‘Never’. Regarding variables on PrEP use, the type of PrEP use was grouped into a binary variable with the categories ‘Daily’ and ‘On demand/intermittent’. The duration of PrEP use was grouped into categories of < 3 months and ≥ 3 months to distinguish short-term PrEP users from PrEP users who obtained repeat prescriptions.

The reasons for stopping PrEP were grouped as follows: The category ‘Reduced need for PrEP’ contained anyone with a positive answer to ‘Partner situation changed’ and ‘Other prevention strategies are sufficient’. The item ‘Partner situation changed’ included anyone with a positive answer to ‘I have fewer partners’, ‘I don’t have sex currently’, and ‘I trust my partner/my partners’. The category ‘Logistic reasons’ contained anyone with a positive answer to ‘Difficulties obtaining PrEP’ and ‘PrEP is unaffordable’. The item ‘Difficulties obtaining PrEP’ included anyone with a positive answer to ‘I cannot get PrEP through my original source any more’ and ‘I have problems finding a doctor willing to prescribe PrEP (only asked in wave 2)’. The category ‘Negative attitudes towards PrEP’ included anyone with a positive answer to ‘I’m afraid of stigma against PrEP users’ and ‘I think that using PrEP is immoral and/or irresponsible’. The category ‘Reservations against characteristics of PrEP’ included anyone with a positive answer to ‘I’m worried about long-term side effects’, ‘I don’t feel adequately protected with PrEP’ (only wave 2), ‘I don’t want to take a daily pill’, and ‘I don’t want to unnecessarily expose my body to chemicals’. The category ‘Side effects’ contained anyone with a positive answer to ‘I experienced side effects’. The category ‘Biological reasons’ included anyone with a positive answer to ‘I had too many STIs’ and ‘I tested positive for HIV’.

### Selection of participants

Participants could enter both study waves. Since the survey was anonymous, we asked participants in the second wave whether they had already participated in wave 1. To ensure that any person would be included in the analysis only once, we excluded participants from wave 2 if they indicated that they had already participated in wave 1.

Since self-reported side effects were only collected in wave 2 of the study, we included all participants from wave 2 into the analysis of side effects irrespective of their participation in wave 1.

### Statistical analysis

Categorical variables are shown as absolute numbers and proportions. Differences in the distribution of categorical variables were analysed with χ^2^ tests. Continuous variables are displayed with medians and interquartile ranges (IQRs). Differences between medians were analysed with a Wilcoxon rank sum test.

Univariable and multivariable logistic regressions were used to analyse factors associated with former PrEP use. Pre-determined co-variables for regression analyses were age, gender, country of origin, annual gross income, satisfaction with sex life, duration of PrEP use, type of PrEP use, number of anal sex partners within the previous 6 months, and condom use. Participants with data missing for any of these variables were excluded from the regression analyses. Interactions were assessed by displaying stratum-specific odds ratios and Likelihood Ratio tests.

### Sensitivity analysis

We stratified the analyses by duration of PrEP use, by type of PrEP use, and by sexual behaviour after stopping PrEP to investigate potentially different patterns of stopping PrEP.

In another analysis, we excluded participants with information missing on whether they had participated in wave 1. In addition, we performed the logistic regression analyses including patients with missing data to test the robustness of the estimates from our complete case analysis.

## Results

We recruited 2337 participants in wave 1 of the study (July–October 2018) and 3484 in wave 2 (April–June 2019; Additional file 1: Appendix S2). We eliminated 364 participants from wave 2 since they had also participated in wave 1. The final sample included 4848 current (wave 1: 2118; wave 2: 2730) and 609 former PrEP users (wave 1: 219, wave 2: 390; Table 1, Additional file 1: Appendix S3).

**Table 1:** Comparison of current and former PrEP users (*n* = 5457)

	Current PrEP users, n (%)	Former PrEP users, n (%)	Univariable Analysis^a^		Multivariable Analysis^b^	
			OR (95% CI)	*p*-value^c^	OR (95% CI)	*p*-value^c^
**Total (n)**	4848	609				
**Age (years)**						
Median (IQR)	37 (30–45)	33 (27–41)				
18–29, n (%)	967 (19.9%)	209 (34.3%)	1.9 (1.4–2.6)	< 0.001	1.6 (1.1–2.3)	0.007
30–39, n (%)	1620 (33.4%)	176 (28.9%)	1		1	
40–49, n (%)	1199 (24.7%)	109 (17.9%)	0.9 (0.7–1.3)	0.639	1.0 (0.7–1.4)	0.850
50–80, n (%)	597 (12.3%)	53 (8.7%)	1.0 (0.7–1.5)	0.995	1.0 (0.6–1.5)	0.839
Missing, n (%)	465 (9.6%)	62 (10.2%)	–			
**Gender, n (%)**						
Male	4299 (88.7%)	537 (88.2%)	1		1	
Female	2 (0.0%)	0 (0.0%)	–			
Transgender / Non-Binary	52 (1.1%)	4 (0.7%)	0.8 (0.2–3.6)	0.822	0.6 (0.1–2.8)	0.499
Intersex	12 (0.2%)	3 (0.5%)	6.2 (1.0–37.3)	0.046	7.4 (1.1–52.2)	0.044
Missing	483 (10.0%)	65 (10.7%)	–			
**Country of origin, n (%)**						
Germany	2531 (52.2%)	325 (53.4%)	1		1	
Outside Germany	813 (16.8%)	119 (19.5%)	1.1 (0.9–1.5)	0.394	0.9 (0.6–1.2)	0.393
Missing	1504 (31.0%)	165 (27.1%)	–			
**Annual gross income, n (%)**						
< 30,000 €	1005 (20.7%)	181 (29.7%)	1		1	
30,000–49,000 €	1104 (22.8%)	132 (21.7%)	0.8 (0.6–1.1)	0.109	1.0 (0.7–1.5)	0.854
≥ 50,000 €	1441 (29.7%)	157 (25.8%)	0.8 (0.6–1.1)	0.252	1.2 (0.9–1.8)	0.231
Missing	1298 (26.8%)	139 (22.8%)	–			
**Satisfaction with sex life, n (%)**						
Happy	3144 (64.9%)	270 (44.3%)	1		1	
Unhappy	298 (6.1%)	111 (18.2%)	4.4 (3.3–5.9)	< 0.001	2.9 (2.0–4.0)	< 0.001
Missing	1406 (29.0%)	228 (37.4%)	–			
**Duration of PrEP use, n (%)**						
< 3 months	1020 (21.0%)	250 (41.1%)	2.5 (1.9–3.2)	< 0.001	1.6 (1.2–2.1)	0.001
≥ 3 months	3214 (66.3%)	275 (45.2%)	1		1	
Missing	614 (12.7%)	84 (13.8%)	–			
**Type of PrEP use, n (%)**						
Daily	2952 (60.9%)	245 (40.2%)	1		1	
On demand/intermittent	1302 (26.9%)	276 (45.3%)	2.6 (2.0–3.3)	< 0.001	1.9 (1.4–2.5)	< 0.001
Missing	594 (12.3%)	88 (14.4%)				
**Number of anal sex partners within the previous 6 months, n (%)**						
0–3	622 (12.8%)	167 (27.4%)	4.8 (3.5–6.5)	< 0.001	2.1 (1.4–2.9)	< 0.001
4–10	1355 (27.9%)	181 (29.7%)	1.8 (1.4–2.5)	< 0.001	1.1 (0.8–1.6)	0.476
> 10	2089 (43.1%)	161 (26.4%)	1		1	
Missing	782 (16.1%)	100 (16.4%)	–			
**Condom use while taking PrEP / since stopping PrEP, n (%)**						
In about half the time/sometimes/never	3164 (65.3%)	160 (26.3%)	1		1	
Always/Often	874 (18.0%)	328 (53.9%)	8.8 (6.7–11.5)	< 0.001	6.7 (5.1–8.9)	< 0.001
Missing	810 (16.7%)	121 (19.9%)	–			
**Recruited through, n (%) [multiple responses possible]**						
Dating Apps	3064 (63.2%)	455 (74.7%)	–			
Community Website	262 (5.4%)	11 (1.8%)	–			
Anonymous Checkpoint	95 (2.0%)	6 (1.0%)	–			
Friends	404 (8.3%)	40 (6.6%)	–			
Missing	1216 (25.1%)	118 (19.4%)	–			

^a^Univariable logistic regression model, ^b^ Multivariable logistic regression model adjusting for age, gender, country of origin, income, satisfaction with sex life, duration of PrEP use, type of PrEP use, partner numbers and condom use. Two thousand six hundred and seventy current and 288 former PrEP users were included in the uni- and multivariable models. ^c^ Wald test. *CI* confidence Interval, *IQR* interquartile range, *OR* odds ratio, *PrEP* pre-exposure prophylaxis

### Factors associated with former PrEP use

Former PrEP users were more often recruited via dating apps (74.7% vs. 63.2%, p < 0.001) (Table 1). In the multivariable model, former PrEP users were more likely than current PrEP users to be aged 18–29 years (adjusted odds ratio (aOR) = 1.6, 95% CI 1.1–2.3) (Table 1). Moreover, they were more likely to have used PrEP for < 3 months (aOR = 1.6, 95% CI 1.2–2.1) and on demand/intermittently (aOR = 1.9, 95% CI 1.4–2.5). Former on-demand/intermittent PrEP use was more pronounced in participants who used PrEP for < 3 months (aOR = 3.1, 95% CI 2.0–4.9) compared to participants who used PrEP for a longer time (aOR = 1.4, 95% CI 1.0–2.0, p_interaction_ = 0.007; Additional file 1: Appendix S4).

Former PrEP users were more unhappy with their sex lives (Table 1) than were current PrEP users, which was more pronounced in participants with daily PrEP use (aOR = 4.5, 95% CI 2.9–7.1) than with on-demand/intermittent use (aOR = 1.8, 95% CI 1.1–2.9, p_interaction_ = 0.005).

Moreover, former PrEP users reported fewer partners within the previous 6 months than did current users (Table 1). They were also more likely to use condoms always/often (aOR = 6.7, 95% CI 5.1–8.9). However, 18.7% of former users indicated using condoms half the time or less during sex and reported having ≥4 anal/vaginal sex partners within the previous 6 months.

Stratification of the results by study wave (Additional file 1: Appendix S3) and excluding participants from wave 2 with information missing on whether they had participated in wave 1 (Additional file 1: Appendix S5) yielded comparable results. A comparison of participants that were included and excluded in the logistic regression model showed that the distribution of the variables were comparable between the groups where information was available (Additional file 1: Appendix S10). Regression analyses including patients with missing data yielded similar results (Additional file 1: Appendix S11).

### Reasons for stopping PrEP

Former PrEP users indicated most commonly a diminished need for PrEP as a reason for stopping, e.g., due to changed partner situations (31.7%) or because other prevention strategies were considered sufficient (24.6%, Table 2).

**Table 2:** Reasons for stopping PrEP in former PrEP users, multiple answers allowed

Reasons for Stopping PrEP	All Participants (***n*** = 609)	Wave 1 (***n*** = 219)	Wave 2 (***n*** = 390)
**Reduced need for PrEP**	**299 (49.1%)**	**112 (51.1%)**	**187 (47.9%)**
Partner situation changed	193 (31.7%)	71 (32.4%)	122 (31.3%)
Other prevention strategies sufficient	150 (24.6%)	53 (24.2%)	97 (24.9%)
**Logistic reasons**	**191 (31.4%)**	**62 (28.3%)**	**129 (33.1%)**
Difficulties obtaining PrEP^a^	72 (11.8%)	21 (9.6%)	51 (13.1%)
PrEP is unaffordable	159 (26.1%)	53 (24.2%)	106 (27.2%)
**Negative attitudes towards PrEP**	**48 (7.9%)**	**17 (7.8%)**	**31 (7.9%)**
Afraid of stigma for taking PrEP	14 (2.3%)	7 (3.2%)	7 (1.8%)
Thinking taking PrEP is immoral	36 (5.9%)	11 (5.0%)	25 (6.4%)
**Reservations against characteristics of PrEP**	**262 (43.0%)**	**81 (37.0%)**	**181 (46.4%)**
Fear of long-term side effects	147 (24.1%)	43 (19.6%)	104 (26.7%)
Do not feel adequately protected by PrEP^b^	50 (8.2%)	–	50 (12.8%)
Not wanting to take a daily pill	142 (23.3%)	40 (18.3%)	102 (26.2%)
Not wanting to take a chemical substance	150 (24.6%)	55 (25.1%)	95 (24.4%)
**Experiencing side effects**	**106 (17.4%)**	**41 (18.7%)**	**65 (16.7%)**
**Biological reasons**	**86 (14.1%)**	**26 (11.9%)**	**60 (15.4%)**
Contracted too many STIs	67 (11.0%)	20 (9.1%)	47 (12.1%)
Positive HIV test	21 (3.4%)	8 (3.7%)	13 (3.3%)
**Other Reasons**	**36 (5.9%)**	**14 (6.4%)**	**22 (5.7%)**
**Missing**	**69 (11.3%)**	**32 (14.6%)**	**37 (9.5%)**

^a^The item ‘I have problems finding a doctor willing to prescribe PrEP’ included in this category was only available in wave 2 of the study. ^b^This was only included in wave 2 of the study. *PrEP* pre-exposure prophylaxis, *STI* sexually transmitted infection

Other reasons reflected reservations against the characteristics of PrEP with TDF/FTC, including not wanting to take a chemical substance (24.6%), fear of long-term side effects (24.1%), and not wanting to take a daily pill (23.3%). Logistic barriers such as difficulties of obtaining PrEP (11.8%) or difficulties of affording PrEP (26.1%) were also common. In wave 2, 10.4% (43/413) indicated that difficulty in finding a doctor to prescribe PrEP was a reason for stopping PrEP.

Almost one in five former PrEP users indicated experiencing side effects as a reason for stopping PrEP (Table 2). Other biological reasons included contracting too many sexually transmitted infections (STIs) during PrEP use (11.0%) and testing positive for HIV (3.4%). Since we do not have information on the status of PrEP use at the time of infection, we are not able to infer PrEP efficacy from these data.

Former users with PrEP use of < 3 months indicated different reasons for stopping PrEP from those of former users who used PrEP for longer (Additional file 1: Appendix S6). Short-term users were more likely to have stopped PrEP due to concerns over long-term side effects (32.0% vs. 22.5%, *p* = 0.015) and not wanting to take a chemical substance (33.2% vs. 24.0%, *p* = 0.020). In contrast, longer-term users more often indicated that their partner situation had changed (40.4% vs. 31.2%, *p* = 0.029).

In addition to being more unhappy with their sex lives, former daily PrEP users were also more likely to indicate side effects as a reason for stopping PrEP (27.8% vs. 12.7%, *p* < 0.001) compared to former on-demand users (Additional file 1: Appendix S7). Conversely, they less often indicated stopping PrEP because they deemed other prevention strategies to be sufficient (22.4% vs. 32.4%, *p* = 0.012).

Former PrEP users reporting ≥4 anal/vaginal sex partners within the previous 6 months and inconsistent condom use were less likely than other former users to state that they considered other prevention strategies to be sufficient (9.6% vs. 28.1%, *p* < 0.001; Additional file 1: Appendix S8). Moreover, they were more likely to indicate logistic reasons for stopping PrEP (46.5% vs. 27.9%, *p* < 0.001).

### Self-reported side effects

We included 2675 participants from wave 2 in our analysis of possible side effects (Additional file 1: Appendix S9). This sample included 363 participants who indicated that they had already participated in wave 1. Interestingly, 26.5% of 2330 current and 44.1% of 345 former PrEP users indicated experiencing side effects of PrEP (*p* < 0.001; Table 3).

**Table 3:** Self-reported side effects in current and former PrEP users^a^ (*n* = 2675)

**Side Effects Ever Experienced**			
	**Current PrEP users (*n*** **= 2330)**	**Former PrEP users who stopped due to side effects (*n*** **= 66)**	**Former PrEP users who did not stop due to side effects (*n*** **= 279)**
Diarrhoea	283 (12.1%)	23 (34.8%)	28 (10.0%)
Nausea	198 (8.5%)	34 (51.5%)	26 (9.3%)
Vomiting	30 (1.3%)	9 (13.6%)	2 (0.7%)
Stomach pain	153 (6.6%)	24 (36.4%)	16 (5.7%)
Headache	140 (6.0%)	30 (45.5%)	30 (10.8%)
Dizziness	119 (5.1%)	16 (24.2%)	17 (6.1%)
Insomnia	150 (6.4%)	23 (34.8%)	27 (9.7%)
Rash	71 (3.0%)	16 (24.2%)	12 (4.3%)
Altered blood values	27 (1.2%)	13 (19.7%)	8 (2.9%)
Other	77 (3.3%)	9 (13.6%)	10 (3.6%)
**Side Effects Currently Experienced**			
	**Current PrEP users (n = 2330)**	**Former PrEP users who stopped due to side effects (*n*** **= 66)**	**Former PrEP users who did not stop due to side effects (*n*** **= 279)**
Diarrhoea	50 (2.1%)	1 (1.5%)	2 (0.7%)
Nausea	35 (1.5%)	1 (1.5%)	2 (0.7%)
Vomiting	7 (0.3%)	–	–
Stomach pain	31 (1.3%)	1 (1.5%)	4 (1.4%)
Headache	33 (1.4%)	5 (7.6%)	8 (2.9%)
Dizziness	36 (1.5%)	4 (6.1%)	1 (0.4%)
Insomnia	60 (2.6%)	7 (10.6%)	9 (3.2%)
Rash	24 (1.0%)	6 (9.1%)	3 (1.1%)
Altered blood values	8 (0.3%)	4 (6.1%)	2 (0.7%)
Other	31 (1.3%)	2 (3.0%)	3 (1.1%)

^a^Only users participating in wave 2 of the survey. *PrEP* pre-exposure prophylaxis

The side-effect profiles of current and former PrEP users who did not stop PrEP owing to side effects, were comparable, except that headache (10.8% vs. 6.0%, *p* = 0.002) and insomnia (9.7% vs. 6.4%, *p* = 0.042) were more common in former users. Former PrEP users who stopped due to side effects most commonly reported nausea, headache and stomach pain. At the time of the survey, most side effects had subsided both in current and former PrEP users (Table 3). However, persisting headache, dizziness, insomnia, rash, or altered blood values were more common in former PrEP users who stopped PrEP due to side effects compared to other former or current PrEP users. In contrast, persisting gastrointestinal side effects were uncommon in all three groups.

## Discussion

In our study we identified several aspects that are relevant for understanding what influences PrEP persistence. Former PrEP users were often younger and some reasons for stopping PrEP were modifiable barriers, e.g., logistic difficulties. Notably, 17.4% of former PrEP users stopped taking PrEP because of side effects. After stopping PrEP, 18.7% of former PrEP users reported high partner numbers and inconsistent condom use. This group often indicated logistic problems as reasons for stopping PrEP and were less likely to stop PrEP because they considered other prevention strategies to be sufficient.

Participants aged 18–29 years were more likely to be found among former PrEP users in our study (aOR = 1.6, 95% CI 1.1–2.3), which is in accordance with results from a single-centre study showing that age < 30 years was associated with PrEP discontinuation (adjusted hazard ratio (aHR) = 2.0, 95% CI 1.4–2.9) [24], and an analysis of US insurance data showing that being aged < 24 years was associated with non-persistence of PrEP (aHR = 2.4, 95%CI 2.1–2.7) [25]. The incidence of new HIV diagnoses among young MSM is high and PrEP programmes should be adapted to fit their needs [26–28]. Studies among young MSM identified keeping up with doctor’s appointments, taking daily medications, fear of side effects and low risk perception as important reasons for PrEP discontinuation or non-uptake in this group [19, 29, 30]. Since low PrEP persistence appears to be a common feature among young MSM across healthcare systems, more qualitative research into the reasons for lower persistence and possible adaptations or alternatives to continuous PrEP use is warranted for this group. This must also include exploration of their sexual needs and expectations, since being unhappy with their sex life was more pronounced in former daily PrEP users. One hypothesis based on these findings could be that people using daily PrEP may start relying on it more often than people who use PrEP on demand or intermittently. Consequently, stopping PrEP may have a bigger impact on their sex lives than people using on demand/intermittent PrEP. This hypothesis should be investigated in future studies.

Former daily PrEP users also more often indicated biological reasons for stopping PrEP, which indicates that some of these former PrEP users may have had to stop PrEP involuntarily. Other compounds and formulations currently under clinical investigation may overcome some of these biological barriers, e.g., because of a different side-effect spectrum or because they could be used in people with impaired kidney function [31, 32].

In our sample, 49.1% of the former PrEP users indicated that a reduced need for PrEP was a reason for stopping it. This is in accordance with findings from other studies of former PrEP users, where about half of the participants indicated stopping PrEP due to a perceived lower HIV risk [33–35]. This has also been described as ‘seasons of risk’, which suggests that HIV risk can undergo periodic changes over time. This translates into some periods where the use of PrEP is indicated and others where it may be unnecessary [13, 14]. Consequently, this subset of former PrEP users appears to be competent in assessing and managing their HIV risk.

A comprehensive HIV prevention strategy needs to include former PrEP users. 18.7% of former PrEP users reported inconsistent condom use and multiple anal/vaginal sex partners within the previous 6 months. Moreover, they were more likely to indicate access barriers as a reason for stopping PrEP and less likely to state that they found other prevention strategies to be sufficient. While promoting condom use is a sensible strategy for all groups at risk, it may be less successful for this group. Additional prevention strategies may include choosing a) HIV-negative partners who use PrEP, b) HIV-positive partners on antiretroviral therapy whose viral load is undetectable, and c) avoiding high-risk practices, e.g. chemsex [36]. However, the applicability and safety of these recommendations need to be investigated quantitatively.

Other reasons for stopping PrEP identified modifiable barriers. Financial burden and lack of health insurance cover have been identified as reasons for stopping PrEP [33–35] and in our sample 26.1% of former participants indicated that PrEP had become unaffordable. Since September 2019, about 3 months after the data collection for this analysis stopped, the costs for PrEP were covered by statutory health insurance in Germany when prescribed by HIV specialists. Therefore, we assume that costs are now less of a factor in Germany. However, this barrier may remain important for people who do not have access to a physician allowed to prescribe PrEP in their area. They may still have to rely on prescriptions with self-payment or on informal sources. Moreover, even though Germany has universal health coverage [37], vulnerable groups such as homeless people or undocumented migrants may not have access to health insurance.

Not wanting to take a daily pill was indicated by 23.3% of the participants as a reason for stopping PrEP. While the current guidelines in Germany endorse only daily PrEP use, other studies have found that PrEP also has a positive efficacy and safety profile when taken ‘on demand’ [12, 38]. It would be interesting to investigate whether on-demand PrEP may be a suitable option for people who are unwilling to take a daily pill. Moreover, other forms of administration, e.g., injectable PrEP or implants, are currently under development and may be more suitable for this group [32]. Alternatively, post-exposure prophylaxis in-pocket (PiP) may be a suitable alternative for people with low-frequency high-risk encounters [39].

About one in four of the former PrEP users indicated stopping PrEP due to fear of long-term side effects. Concern about long-term toxicity of TDF/FTC has also been identified as a barrier in other studies [33, 40]. However, a meta-analysis of randomised controlled trials on PrEP came to the conclusion that renal abnormalities usually resolved quickly after stopping PrEP, or even during PrEP use, and that serious renal adverse events were rare [15]. Moreover, this analysis found no difference in risk of bone fractures. Thus, balanced information on the actual risks and benefits of PrEP should be communicated effectively to potential PrEP users so that they may make informed decisions.

The spectrum of self-reported side effects in people taking PrEP under real-life conditions was similar to that previously described in clinical trials [41]. Interestingly, former on-demand users were less likely to stop PrEP owing to side effects. Thus, taking PrEP on demand may be a potential strategy to minimise side-effect risks. Most reported side effects were transient and had already dissipated at the time of the survey. Consequently, effective information on side-effect management should be available to all PrEP users to avoid unnecessary PrEP terminations due to transient and manageable side effects. Former users who stopped due to side effects were more likely to report that they still experienced headache, dizziness, insomnia, rash or altered blood values at the time of the survey compared to other participants. Notably, this did not extend to gastrointestinal side effects such as diarrhoea, nausea or vomiting. From our data we cannot infer whether these conditions were exacerbated by PrEP or whether they developed as a cause of PrEP. Clinical investigations should investigate the significance of these findings.

Our study has several strengths. We recruited a large number of current and former PrEP users, which overcame the limitations of previous studies with small samples and allowed us to analyse different subgroups of PrEP users with satisfactory statistical precision. In addition, we recruited PrEP users independent of the location where they received or used to receive their PrEP, thereby avoiding selection bias of people receiving PrEP under medical supervision.

Some limitations need to be considered. We do not know how applicable the results are to PrEP users not using online resources. However, since internet use and dating apps are widely used among MSM, we do not consider this to have a major influence [42]. Moreover, we could not gather data on non-participation to investigate potential selection bias. The analyses are based on self-reported data from the participants, which may have led to more socially acceptable answers on sensitive topics, e.g. condom use. However, since many indicated low condom use, we do not expect this to have a major impact. On-demand/intermittent PrEP users might have misclassified themselves into the group of former PrEP users if they were currently not using PrEP. This would have made the groups more similar and biased our results towards the null. The participants could also indicate several reasons for stopping PrEP, so it is difficult to estimate the net effect of removing individual barriers. However, since decision-making is multifactorial, we decided to allow recording multiple reasons per person so that we would be able to report on the spectrum of reasons for stopping and all potential barriers.

We used the full case analysis approach for our regression models and had to exclude participants with missing information on any of the included variables. While we tried to motivate all participants to complete the survey with a lottery gift voucher as incentive, some participants left the survey before completion. Thus, questions at the end of the survey automatically had a higher proportion of missing values. While the remaining analytical sample had sufficient statistical power for our regression analyses, the generalisability of the results may be limited. However, we could not find a pattern regarding age, gender identity, sexual behaviour or PrEP use that would indicate a differential dropout of some subgroups from the survey and thus might have led to the underrepresentation of these subgroups in our analysis. It is, however, possible that subgroups defined by variables not measured in this survey may have differentially dropped out.

The side effects were self-reported and are thus subject to the participants’ awareness. While underreporting of noticeable side effects such as diarrhoea seems unlikely, the participants’ awareness of non-acute side effects depends on their communication with the physician and potentially on the frequency of medical testing. Thus, non-acute side effects may be underreported in this survey. In contrast, participants may perceive unrelated events as associated with PrEP use. Thus, the symptomatic side effects reported in this study may be overestimates. However, since the frequencies of side effects were similar to those from the product information, we do not expect these factors to have a major effect. In addition, we were not able to determine from our data the causality between side effects and PrEP use.

## Conclusions

Stopping PrEP can be part of a natural fluctuation of individual HIV risk, but it can also be caused by avoidable factors. To maximise persistence with PrEP we suggest a) to develop strategies to enhance persistence of younger PrEP users, b) to reduce financial and logistic barriers, e.g., through health insurance cover, and c) to ensure effective communication on mitigating potential short-term and long-term toxicities. Moreover, the feasibility and effectiveness of HIV prevention strategies for people who stopped PrEP use need to be the focus of future studies.

## Supplementary Information

**Additional file 1.**

## Data Availability

The datasets generated during and analysed during the current study are available from the corresponding author on reasonable request.

## Abbreviations

aHR: Adjusted Hazard Ratio
aOR: Adjusted Odds Ratio
CI: Confidence interval
IQR: Interquartile range
MSM: Men, who have sex with men
OR: Odds ratio
PiP: Post-exposure prophylaxis in-pocket
PrEP: Pre-exposure prophylaxis
STI: Sexually transmitted infection
TDF/FTC: Tenofovirdisoproxil/Emtricitabine
