# Supplementary material for: Barriers to using HIV pre-exposure prophylaxis (PrEP) and sexual behaviour after stopping PrEP: a cross-sectional study in Germany
Source: BMC Public Health. 2021 Jan 19;21:159. doi: 10.1186/s12889-021-10174-4 (PMC7816315; doi:10.1186/s12889-021-10174-4)
Supplement: Supplementary file 1 — Additional file 1. [file 12889_2021_10174_MOESM1_ESM.docx]

# Appendix: Barriers to using HIV Pre-exposure prophylaxis (PrEP) and sexual behaviour after stopping PrEP: a cross-sectional study in Germany

# Overview

Appendix S1: Survey questions

Appendix S2: Participant selection for analysis:

Appendix S3: Comparison of current and former PrEP users by study waves

Appendix S4: Comparison of current PrEP and former PrEP users according to duration of PrEP use

Appendix S5: Comparison of current PrEP and former PrEP users excluding participants from wave 2 if they have information missing as to whether they had participated in wave 1

Appendix S6: Reasons for stopping PrEP in former PrEP users stratified by duration of PrEP use

Appendix S7: Reasons for stopping PrEP in former PrEP users stratified by type of PrEP use

Appendix S8: Reasons for stopping PrEP in former PrEP users stratified by sexual behaviour

Appendix S9: Participants in wave 2 who reported information on possible side effects (including those who indicated participating in wave 1)

Appendix S10: Comparison of participants, who are in- and excluded in the logistic regression analysis

Appendix S 11: Sensitivity analysis of multivariable logistic regression model including missing values as a separate category

Appendix S1: Survey questions

The questions from wave 1 of the study have been published in the Appendix S1 of:

“Factors associated with the informal use of HIV pre-exposure prophylaxis in Germany: a cross-sectional study.” Koppe U, Marcus U, Albrecht S, Jansen K, Jessen H, Gunsenheimer-Bartmeyer B, Bremer V.; J Int AIDS Soc. 2019 Oct;22(10):e25395. doi: 10.1002/jia2.25395.

These questions were also used in wave 2 with the following exceptions:

| **General information – Current and former PrEP users** | |
| --- | --- |
| What gender do you identify with? | - Male - Female - Trans male - Trans female - Inter - Non-binary - Other: |
| What gender were you assigned at birth? | - Male - Female - Intersexual |
| What is your approximate gross income per year?  *Gross income: income before deduction of taxes and social security contributions* | - Less than 30,000€ - 30,000–39,000€ - 40,000–49,000€ - 50,000–59,000€ - 60,000–69,000€ - 70,000€ or more - I don’t have any income / I am unemployed - I don’t know |
| Have you participated in this survey before?  (e.g. between July and October 2018) | - Yes - No - I don’t remember |
| What are the first three digits of the postal code of your residence in Germany?  If you are not living in Germany please indicate the first three digits of the postal code of the place where you are predominantly staying (e.g., hotel).  *Examples:*  *If your postal code is 04103, please type 041.*  *If your postal code is 10777, please type 107.* | |

| **PrEP use and sexual behaviour – Current PrEP users** | |
| --- | --- |
| Why do you take PrEP? | Select all that apply:   - I don’t want to use condoms. - I am allergic to condoms. - My partner doesn’t want to use condoms. - Sex without a condom is expected by my peers. - I want to protect myself against HIV in case the condom breaks. - Sometimes condoms aren’t available. - It’s more convenient since I don’t have to talk about or negotiate condom use. - I don’t trust my partner/s that they reliably use condoms or PrEP. - I cannot get an erection or an orgasm when I use a condom. - I am in an open relationship and I want to protect my partner. - My partner is HIV positive. - I use condoms and I want additional protection. - I am less anxious during sex. - Other: _________ |
| We now have a few more questions about your sex life:  With how many different partners have you had anal and/or vaginal sex within the last 6 months? | - 0 - 1 - 2–3 - 4–5 - 6–10 - 11–20 - more than 20 - I don’t know |
| Has the number of sexual partners or the number of sexual contacts increased since / when you are taking PrEP? | - Yes, I am having more partners - Yes, I am having more sexual contacts - Yes, I am having more partners and more contacts - No - I don’t know |
| How often do you use condoms for anal / vaginal sex in periods when you are taking PrEP? | - Always - Often - About half of the times I have anal sex - Sometimes - Never - I don’t know |
| Since I am taking PrEP, I am using condoms for anal / vaginal sex… | - More often than before - As often as before - Less often than before - I have stopped using condoms altogether - I don’t know |
| Where did you obtain PrEP from?  (please indicate the last source where you obtained PrEP) | - German pharmacy – prescription for about 40–50€ (blister prescription) - German pharmacy prescription for more than 50€, but less than 100€ - German pharmacy – prescription for 500–800€ - German pharmacy – my health insurance covers the costs - Friends - Dealer - Sex Party - Research Study - Internet / ordered online from another country - I regularly travel to another country where I get PrEP - I used PEP-medication as PrEP - Other: _________ |
| Why were you not tested before starting PrEP?  Select all that apply: | - I couldn’t afford the tests - The tests were not offered to me - I didn’t have time to take the tests - I didn’t want to take the tests - I didn’t think I would benefit from these tests - I didn’t know I was supposed to take the tests - Other reason: |
| Have you been tested for HIV, other sexually transmitted infections (STI), or have been checked for your kidney function **while taking PrEP?** | - Yes (all or some of the tests) - No - I don’t know |
| Why are you not getting tested while taking PrEP?  Select all that apply: | - I can’t afford the tests - The tests were not offered to me - I don't have time to take the tests - I don't want to take the tests - I don't think I would benefit from these tests - I didn’t know I was supposed to take the tests - Other reason: |
| Where do you get tested while you are using PrEP (e.g. for HIV, STIs or kidney function)?  Select all that apply: | - At the doctor who prescribes my PrEP - At a doctor who does not prescribe my PrEP - At a community-based testing site / anonymous testing clinic - Self-testing - Other: ______ |
| Are you currently experiencing side effects caused by PrEP or have you experienced side effects in the past? | - Yes - No - I don’t know |
| Which side effects have you experienced or were you diagnosed with?  Select all that apply: | - Diarrhoea - Nausea - Vomiting - Stomach ache - Headache - Dizziness - Insomnia / abnormal dreams - Skin rash / allergic reaction - Altered blood parameters - Other: |
| Are you currently experiencing the side effects or have they subsided? | - Is still going on - Has subsided |

| **PrEP and sexual behaviour – Former PrEP users** | |
| --- | --- |
| Why did you stop using PrEP?  Select all that apply: | - I feel safe enough with other prevention strategies (condoms, etc). - I don’t need it because I am currently not having sex. - I feel I don’t need it because I have fewer sexual partners than before. - I feel I don’t need it anymore because I trust my partner / partners. - I don’t want to take a daily pill. - I experienced side effects. - I am worried about long-term side effects with PrEP. - I had too many STIs while I was having condomless sex on PrEP. - The price for PrEP and/or the medical tests is too high for me. - I cannot get PrEP any more through my original source (e.g. clinical trial ended, dealer changed, friends do not share PrEP any more). - I have problems finding a doctor willing to prescribe PrEP. - I would like to use PrEP but I’m afraid of stigma against PrEP users by others / my partner. - I tested positive for HIV. - I think that using PrEP is immoral and/or irresponsible. - I don’t want to unnecessarily expose my body to chemicals. - I don’t feel adequately protected with PrEP. - Other. |
| We now have a few more questions about your sex life:  With how many different sexual partners have you had anal/vaginal sex with in the last 6 months? | - 0 - 1 - 2–3 - 4–5 - 6–10 - 11–20 - More than 20 - I don’t know |
| How often do you use condoms to prevent HIV infection during anal/vaginal sex since stopping PrEP? | - Always - Often - In about half of the times I had sex - Sometimes - Never - I don’t know |
| Did you experience side effects caused by PrEP? | - Yes - No - I don’t know |
| Which side effects have you experienced or were you diagnosed with?  Select all that apply: | - Diarrhoea - Nausea - Vomiting - Stomach ache - Headache - Dizziness - Insomnia/abnormal dreams - Skin rash/allergic reaction - Altered blood parameters - Other: |
| Are you still experiencing the side effects or have they subsided? | - Is still going on - Has subsided |

Appendix S2: Participant selection for analysis:


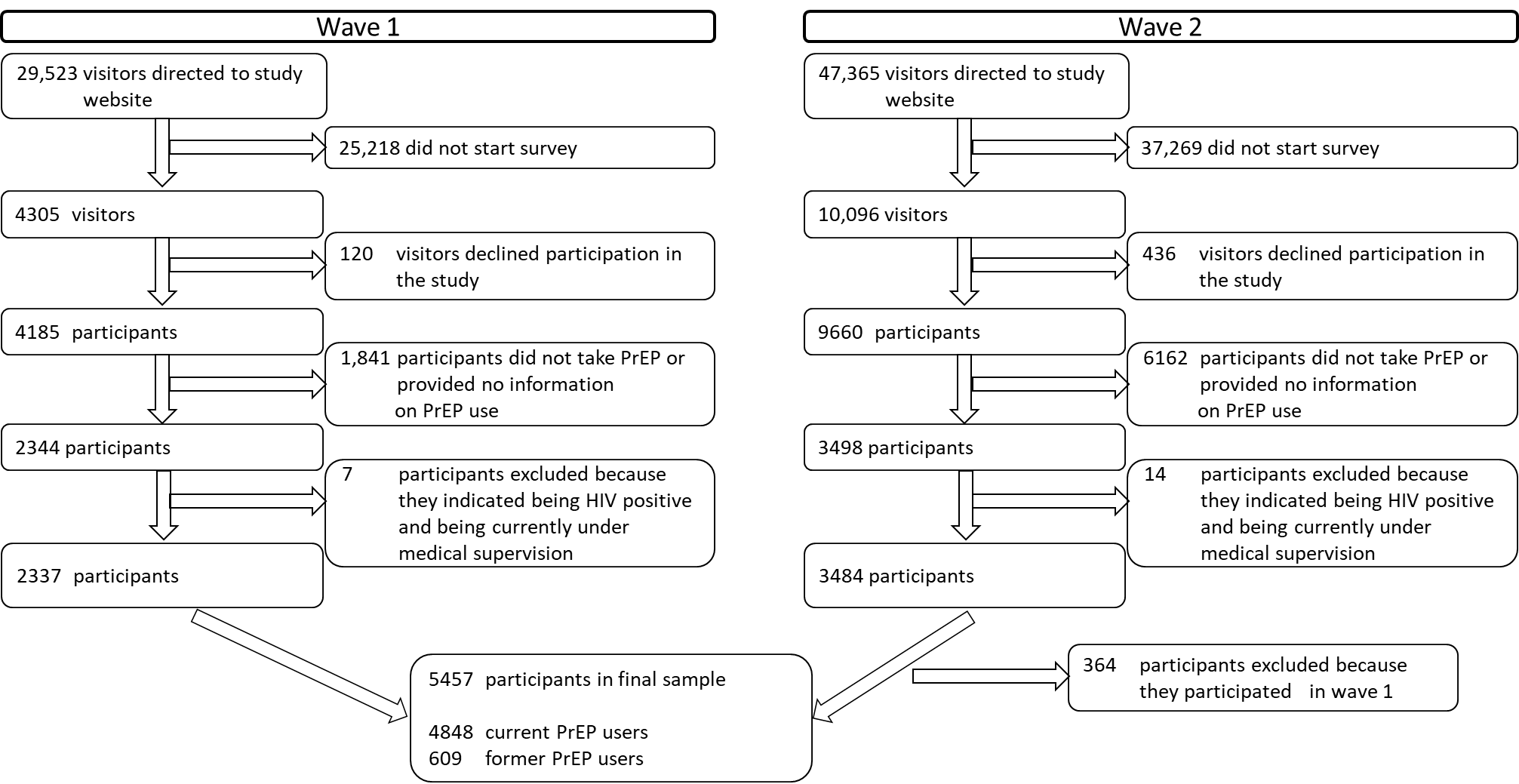


Appendix S3: Comparison of current and former PrEP users by study waves

Wave 1 (n = 2337)

|  | Current PrEP users, n (%) | Former PrEP users, n (%) | Univariable Analysis^ꝉ^ | | Multivariable Analysis^‡^ | |
| --- | --- | --- | --- | --- | --- | --- |
|  |  |  | OR (95% CI) | p-value^§^ | OR (95% CI) | p-value^§^ |
| **Total (n)** | 2,118 | 219 |  |  |  |  |
| **Age (years)** |  |  |  |  |  |  |
| Median (IQR) | 38 (31–45) | 33 (27–41) |  |  |  |  |
| 18–29, n (%) | 341 (16.1%) | 66 (30.1%) | 2.1 (1.3–3.4) | 0.005 | 1.6 (0.9–2.9) | 0.096 |
| 30–39, n (%) | 637 (30.1%) | 59 (26.9%) | 1 |  | 1 |  |
| 40–49, n (%) | 519 (24.5%) | 30 (13.7%) | 0.7 (0.4–1.3) | 0.309 | 0.7 (0.4–1.3) | 0.264 |
| 50–80, n (%) | 232 (11.0%) | 21 (9.6%) | 1.2 (0.6–2.4) | 0.522 | 1.1 (0.6–2.3) | 0.719 |
| Missing, n (%) | 389 (18.4%) | 43 (19.6%) | - |  |  |  |
| **Gender, n (%)** |  |  |  |  |  |  |
| Male | 1712 (80.8%) | 174 (79.5%) | 1 |  | 1 |  |
| Female | - | - |  |  |  |  |
| Transgender/Non-Binary | 12 (0.6%) | 1 (0.5%) | 3.0 (0.3–27.5) | 0.322 | 1.4 (0.1–16.2) | 0.782 |
| Intersex | 4 (0.2%) | 1 (0.5%) | - |  |  |  |
| Missing | 390 (18.4%) | 43 (19.6%) | - |  |  |  |
| **Country of origin, n (%)** |  |  |  |  |  |  |
| Germany | 1158 (54.7%) | 118 (53.9%) | 1 |  | 1 |  |
| Outside Germany | 381 (18.0%) | 34 (15.5%) | 1.0 (0.6–1.6) | 0.914 | 0.7 (0.4–1.3) | 0.264 |
| Missing | 579 (27.3%) | 67 (30.6%) | - |  |  |  |
| **Annual gross income, n (%)** |  |  |  |  |  |  |
| <30,000 € | 419 (19.8%) | 56 (25.6%) | 1 |  | 1 |  |
| 30,000–49,000 € | 539 (25.4%) | 53 (24.2%) | 0.7 (0.4–1.1) | 0.134 | 0.8 (0.4–1.5) | 0.465 |
| ≥50,000 € | 673 (31.8%) | 60 (27.4%) | 0.8 (0.5–1.2) | 0.272 | 1.2 (0.7–2.2) | 0.563 |
| Missing | 487 (23.0%) | 50 (22.8%) | - |  |  |  |
| **Satisfaction with sex life, n (%)** |  |  |  |  |  |  |
| Happy | 1364 (64.4%) | 96 (43.8%) | 1 |  | 1 |  |
| Unhappy | 119 (5.6%) | 34 (15.5%) | 3.5 (2.1–5.8) | <0.001 | 1.8 (1.0–3.3) | 0.040 |
| Missing | 635 (30.0%) | 89 (40.6%) | - |  |  |  |
| **Duration of PrEP use, n (%)** |  |  |  |  |  |  |
| <3 months | 379 (17.9%) | 86 (39.3%) | 2.8 (1.8–4.2) | <0.001 | 1.8 (1.1–2.8) | 0.015 |
| ≥3 months | 1380 (65.2%) | 94 (42.9%) | 1 |  | 1 |  |
| Missing | 359 (16.9%) | 39 (17.8%) | - |  |  |  |
| **Type of PrEP use, n (%)** |  |  |  |  |  |  |
| Daily | 1256 (59.3%) | 80 (36.5%) | 1 |  | 1 |  |
| On demand / intermittent | 504 (23.8%) | 99 (45.2%) | 2.5 (1.7–3.7) | <0.001 | 1.7 (1.1–2.7) | 0.022 |
| Missing | 358 (16.9%) | 40 (18.3%) | - |  |  |  |
| **Number of anal sex partners within the last 6 months, n (%)** | | | | | | |
| 0–3 | 250 (11.8%) | 63 (28.8%) | 5.0 (3.0–8.3) | <0.001 | 2.3 (1.3–4.0) | 0.006 |
| 4–10 | 538 (25.4%) | 57 (26.0%) | 2.0 (1.2–3.2) | 0.007 | 1.2 (0.7–2.0) | 0.507 |
| >10 | 937 (44.2%) | 56 (25.6%) | 1 |  | 1 |  |
| Missing | 393 (18.6%) | 43 (19.6%) | - |  |  |  |
| **Condom use while taking PrEP/since stopping PrEP, n (%)** | | | | | | |
| In about half the  times/sometimes/never | 1380 (65.2%) | 61 (27.9%) | 1 |  | 1 |  |
| Always/Often | 356 (16.8%) | 108 (49.3%) | 9.0 (5.8–13.9) | <0.001 | 6.9 (4.3–11.1) | <0.001 |
| Missing | 382 (18.0%) | 50 (22.8%) | - |  |  |  |
| **Recruited through, n (%) [multiple responses possible]** | | | | | | |
| Dating Apps | 1320 (62.3%) | 158 (72.1%) | - |  |  |  |
| Community Website | 186 (8.8%) | 6 (2.7%) | - |  |  |  |
| Anonymous Checkpoint | 53 (2.5%) | 3 (1.4%) | - |  |  |  |
| Friends | 214 (10.1%) | 18 (8.2%) | - |  |  |  |
| Missing | 443 (20.9%) | 45 (20.5%) | - |  |  |  |

^ꝉ^ Univariable logistic regression model, ^‡^ Multivariable logistic regression model adjusting for age, gender, country of origin, income, satisfaction with sex life, type of PrEP use, partner numbers and condom use. One thousand two hundred and thirty-three current and 102 former PrEP users were included into the uni- and multivariable models. ^§^ Wald test. CI: confidence interval, IQR: interquartile range, OR: odds ratio, PrEP: pre-exposure prophylaxis

Wave 2 (n = 3120)

|  | Current PrEP users, n (%) | Former PrEP users, n (%) | Univariable Analysis^ꝉ^ | | Multivariable Analysis^‡^ | |
| --- | --- | --- | --- | --- | --- | --- |
|  |  |  | OR (95% CI) | p-value^§^ | OR (95% CI) | p-value^§^ |
| **Total (n)** | 2730 | 390 |  |  |  |  |
| **Age (years)** |  |  |  |  |  |  |
| Median (IQR) | 36 (30–45) | 33 (26–41) |  |  |  |  |
| 18–29, n (%) | 626 (22.9%) | 143 (36.7%) | 1.8 (1.3–2.7) | 0.002 | 1.5 (1.0–2.4) | 0.055 |
| 30–39, n (%) | 983 (36.0%) | 117 (30.0%) | 1 |  | 1 |  |
| 40–49, n (%) | 680 (24.9%) | 79 (20.3%) | 1.1 (0.7–1.6) | 0.786 | 1.1 (0.7–1.8) | 0.581 |
| 50–80, n (%) | 365 (13.4%) | 32 (8.2%) | 0.9 (0.5–1.5) | 0.607 | 0.8 (0.4–1.5) | 0.458 |
| Missing, n (%) | 76 (2.8%) | 19 (4.9%) | - |  |  |  |
| **Gender, n (%)** |  |  |  |  |  |  |
| Male | 2587 (94.8%) | 363 (93.1%) | 1 |  | 1 |  |
| Female | 2 (0.1%) | 0 (0.0%) | - |  |  |  |
| Transgender/Non-Binary | 40 (1.5%) | 3 (0.8%) | 0.4 (0.1–3.2) | 0.413 | 0.3 (0.0–2.8) | 0.302 |
| Intersex | 8 (0.3%) | 2 (0.5%) | 7.7 (1.1–55.3) | 0.041 | 14.7 (1.7–123.5) | 0.013 |
| Missing | 93 (3.4%) | 22 (5.6%) | - |  |  |  |
| **Country of origin, n (%)** |  |  |  |  |  |  |
| Germany | 1373 (50.3%) | 207 (53.1%) | 1 |  | 1 |  |
| Outside Germany | 432 (15.8%) | 85 (21.8%) | 1.2 (0.9–1.8) | 0.221 | 0.9 (0.6–1.4) | 0.644 |
| Missing | 925 (33.9%) | 98 (25.1%) | - |  |  |  |
| **Annual gross income, n (%)** |  |  |  |  |  |  |
| <30,000 € | 586 (21.5%) | 125 (32.1%) | 1 |  | 1 |  |
| 30,000–49,000 € | 565 (20.7%) | 79 (20.3%) | 0.9 (0.6–1.3) | 0.471 | 1.3 (0.8–2.0) | 0.294 |
| ≥50,000 € | 768 (28.1%) | 97 (24.9%) | 0.9 (0.6–1.3) | 0.617 | 1.2 (0.8–1.9) | 0.338 |
| Missing | 811 (29.7%) | 89 (22.8%) | - |  |  |  |
| **Satisfaction with sex life, n (%)** |  |  |  |  |  |  |
| Happy | 1780 (65.2%) | 174 (44.6%) | 1 |  | 1 |  |
| Unhappy | 179 (6.6%) | 77 (19.7%) | 5.0 (3.5–7.1) | <0.001 | 3.8 (2.5–5.8) | <0.001 |
| Missing | 771 (28.2%) | 139 (35.6%) | - |  |  |  |
| **Duration of PrEP use, n (%)** |  |  |  |  |  |  |
| <3 months | 641 (23.5%) | 164 (42.1%) | 2.3 (1.7–3.1) | <0.001 | 1.4 (1.0–2.1) | 0.049 |
| ≥3 months | 1834 (67.2%) | 181 (46.4%) | 1 |  | 1 |  |
| Missing | 255 (9.3%) | 45 (11.5%) |  |  |  |  |
| **Type of PrEP use, n (%)** |  |  |  |  |  |  |
| Daily | 1696 (62.1%) | 165 (42.3%) | 1 |  | 1 |  |
| On demand/intermittent | 798 (29.2%) | 177 (45.4%) | 2.7 (1.9–3.6) | <0.001 | 2.0 (1.4–2.9) | <0.001 |
| Missing | 236 (8.6%) | 48 (12.3%) | - |  |  |  |
| **Number of anal sex partners within the last 6 months, n (%)** | | | | | | |
| 0–3 | 372 (13.6%) | 104 (26.7%) | 4.5 (3.0–6.7) | <0.001 | 1.9 (1.2–3.0) | 0.005 |
| 4–10 | 817 (29.9%) | 124 (31.8%) | 1.7 (1.2–2.5) | 0.005 | 1.0 (0.7–1.5) | 0.986 |
| >10 | 1152 (42.2%) | 105 (26.9%) | 1 |  | 1 |  |
| Missing | 389 (14.2%) | 57 (14.6%) | - |  |  |  |
| **Condom use while taking PrEP/since stopping PrEP, n (%)** | | | | | | |
| In about half the  times/sometimes/never | 1784 (65.3%) | 99 (25.4%) | 1 |  | 1 |  |
| Always/Often | 518 (19.0%) | 220 (56.4%) | 8.5 (6.0–11.9) | <0.001 | 7.0 (4.8–10.1) | <0.001 |
| Missing | 428 (15.7%) | 71 (18.2%) | - |  |  |  |
| **Recruited through, n (%) [multiple responses possible]** | | | | | | |
| Dating Apps | 1744 (63.9%) | 297 (76.2%) | - |  |  |  |
| Community Website | 76 (2.8%) | 5 (1.3%) | - |  |  |  |
| Anonymous Checkpoint | 42 (1.5%) | 3 (0.8%) | - |  |  |  |
| Friends | 190 (7.0%) | 22 (5.6%) | - |  |  |  |
| Missing | 773 (28.3%) | 73 (18.7%) | - |  |  |  |

^ꝉ^ Univariable logistic regression model, ^‡^ Multivariable logistic regression model adjusting for age, gender, country of origin, income, satisfaction with sex life, type of PrEP use, partner numbers and condom use. One thousand four hundred and thirty-seven current and 186 former PrEP users were included into the uni- and multivariable models. ^§^ Wald test. CI: confidence interval, IQR: interquartile range, OR: odds ratio, PrEP: pre-exposure prophylaxis

Appendix S4: Comparison of current PrEP and former PrEP users according to duration of PrEP use

PrEP use <3 months (n = 1270)

|  | Current PrEP users, n (%) | Former PrEP users, n (%) | Univariable Analysis^ꝉ^ | | Multivariable Analysis^‡^ | |
| --- | --- | --- | --- | --- | --- | --- |
|  |  |  | OR (95% CI) | p-value^§^ | OR (95% CI) | p-value^§^ |
| **Total (n)** | 1020 | 250 |  |  |  |  |
| **Age (years)** |  |  |  |  |  |  |
| Median (IQR) | 35 (29–43) | 32 (26–39) |  |  |  |  |
| 18–29, n (%) | 275 (27.0%) | 102 (40.8%) | 1.6 (1.0–2.6) | 0.041 | 1.5 (0.9–2.6) | 0.123 |
| 30–39, n (%) | 371 (36.4%) | 87 (34.8%) | 1 |  | 1 |  |
| 40–49, n (%) | 235 (23.0%) | 40 (16.0%) | 0.8 (0.5–1.4) | 0.400 | 0.9 (0.5–1.7) | 0.763 |
| 50–80, n (%) | 130 (12.7%) | 17 (6.8%) | 0.7 (0.3–1.4) | 0.271 | 0.7 (0.3–1.7) | 0.481 |
| Missing, n (%) | 9 (0.9%) | 4 (1.6%) | - |  |  |  |
| **Gender, n (%)** |  |  |  |  |  |  |
| Male | 998 (97.8%) | 242 (96.8%) | 1 |  | 1 |  |
| Female | 0 | 0 | - |  |  |  |
| Transgender / Non-Binary | 9 (0.9%) | 2 (0.8%) | 0.7 (0.1–5.9) | 0.759 | 0.9 (0.1–9.1) | 0.936 |
| Intersex | 3 (0.3%) | 2 (0.8%) | - |  |  |  |
| Missing | 10 (1.0%) | 4 (1.6%) | - |  |  |  |
| **Country of origin, n (%)** |  |  |  |  |  |  |
| Germany | 595 (58.3%) | 142 (56.8%) | 1 |  | 1 |  |
| Outside Germany | 182 (17.8%) | 58 (23.2%) | 1.2 (0.8–1.8) | 0.455 | 1.0 (0.6–1.7) | 0.982 |
| Missing | 243 (23.8%) | 50 (20.0%) | - |  |  |  |
| **Annual gross income, n (%)** |  |  |  |  |  |  |
| <30,000 € | 278 (27.3%) | 94 (37.6%) | 1 |  | 1 |  |
| 30,000–49,000 € | 253 (24.8%) | 62 (24.8%) | 0.9 (0.6–1.5) | 0.706 | 1.1 (0.6–2.0) | 0.695 |
| ≥50,000 € | 304 (29.8%) | 61 (24.4%) | 0.8 (0.5–1.3) | 0.407 | 1.1 (0.6–1.9) | 0.811 |
| Missing | 185 (18.1%) | 33 (13.2%) | - |  |  |  |
| **Satisfaction with sex life, n (%)** |  |  |  |  |  |  |
| Happy | 722 (70.8%) | 126 (50.4%) | 1 |  | 1 |  |
| Unhappy | 83 (8.1%) | 52 (20.8%) | 3.4 (2.1–5.6) | <0.001 | 2.0 (1.1–3.5) | 0.021 |
| Missing | 215 (21.1%) | 72 (28.8%) | - |  |  |  |
| **Type of PrEP use, n (%)** |  |  |  |  |  |  |
| Daily | 656 (64.3%) | 84 (33.6%) | 1 |  | 1 |  |
| On demand/intermittent | 362 (35.5%) | 160 (64.0%) | 3.5 (2.3–5.3) | <0.001 | 3.1 (2.0–4.9) | <0.001 |
| Missing | 2 (0.2%) | 6 (2.4%) | - |  |  |  |
| **Number of anal sex partners within the last 6 months, n (%)** | | | | | | |
| 0–3 | 208 (20.4%) | 96 (38.4%) | 4.2 (2.5–6.9) | <0.001 | 2.6 (1.4–4.5) | 0.001 |
| 4–10 | 373 (36.6%) | 81 (32.4%) | 1.7 (1.0–2.8) | 0.036 | 1.2 (0.7–2.1) | 0.561 |
| >10 | 390 (38.2%) | 64 (25.6%) | 1 |  | 1 |  |
| Missing | 49 (4.8%) | 9 (3.6%) | - |  |  |  |
| **Condom use while taking PrEP/since stopping PrEP, n (%)** | | | | | | |
| In about half the  times/sometimes/never | 659 (64.6%) | 67 (26.8%) | 6.6 (4.2–10.2) | <0.001 | 6.0 (3.7–9.7) | <0.001 |
| Always/Often | 294 (28.8%) | 167 (66.8%) | 1 |  | 1 |  |
| Missing | 67 (6.6%) | 16 (6.4%) | - |  |  |  |
| **Recruited through, n (%) [multiple responses possible]** | | | | | | |
| Dating Apps | 720 (70.7%) | 211 (84.4%) | - |  |  |  |
| Community Website | 59 (5.8%) | 4 (1.6%) | - |  |  |  |
| Anonymous Checkpoint | 26 (2.5%) | 1 (0.4%) | - |  |  |  |
| Friends | 92 (9.3%) | 22 (8.8%) | - |  |  |  |
| Missing | 175 (17.2%) | 21 (8.4%) | - |  |  |  |

^ꝉ^ Univariable logistic regression model, ^‡^ Multivariable logistic regression model adjusting for age, gender, country of origin, income, satisfaction with sex life, type of PrEP use, partner numbers and condom use. Six hundred and sixteen current and 123 former PrEP users were included into the uni- and multivariable models. ^§^ Wald test. CI: confidence interval, IQR: interquartile range, OR: odds ratio, PrEP: pre-exposure prophylaxis

PrEP use ≥3 months (n = 1270)

|  | Current PrEP users, n (%) | Former PrEP users, n (%) | Univariable Analysis^ꝉ^ | | Multivariable Analysis^‡^ | |
| --- | --- | --- | --- | --- | --- | --- |
|  |  |  | OR (95% CI) | p-value^§^ | OR (95% CI) | p-value^§^ |
| **Total (n)** | 3,214 | 275 |  |  |  |  |
| **Age (years)** |  |  |  |  |  |  |
| Median (IQR) | 38 (31–45) | 34 (27–43) |  |  |  |  |
| 18–29, n (%) | 633 (19.7%) | 98 (35.6%) | 1.9 (1.2–2.8) | 0.003 | 1.7 (1.1–2.8) | 0.021 |
| 30–39, n (%) | 1191 (37.1%) | 80 (29.1%) | 1 |  | 1 |  |
| 40–49, n (%) | 932 (29.0%) | 63 (22.9%) | 1.1 (0.7–1.6) | 0.808 | 1.0 (0.6–1.6) | 0.976 |
| 50–80, n (%) | 437 (13.6%) | 34 (12.4%) | 1.3 (0.8–2.1) | 0.381 | 1.1 (0.6–2.0) | 0.661 |
| Missing, n (%) | 21 (0.7%) | 0 (0.0%) | - |  |  |  |
| **Gender, n (%)** |  |  |  |  |  |  |
| Male | 3143 (97.8%) | 273 (99.3%) | 1 |  | 1 |  |
| Female | 1 (0.0%) | 0 (0.0%) | - |  |  |  |
| Transgender / Non-Binary | 40 (1.2%) | 1 (0.4%) | 0.8 (0.1–6.3) | 0.860 | 0.4 (0.0–3.9) | 0.458 |
| Intersex | 7 (0.2%) | 1 (0.4%) | 4.1 (0.4–40.3) | 0.218 | 4.2 (0.4–46.8) | 0.246 |
| Missing | 23 (0.7%) | 0 (0.0%) | - |  |  |  |
| **Country of origin, n (%)** |  |  |  |  |  |  |
| Germany | 1936 (60.2%) | 183 (66.5%) | 1 |  | 1 |  |
| Outside Germany | 631 (19.6%) | 61 (22.2%) | 1.1 (0.8–1.6) | 0.625 | 0.8 (0.5–1.3) | 0.364 |
| Missing | 647 (20.1%) | 31 (11.3%) | - |  |  |  |
| **Annual gross income, n (%)** |  |  |  |  |  |  |
| <30,000 € | 727 (22.6%) | 87 (31.6%) | 1 |  | 1 |  |
| 30,000–49,000 € | 851 (26.5%) | 70 (25.5%) | 0.7 (0.5–1.1) | 0.167 | 1.1 (0.7–1.7) | 0.767 |
| ≥50,000 € | 1137 (35.4%) | 96 (34.9%) | 1.0 (0.7–1.4) | 0.858 | 1.5 (0.9–2.3) | 0.107 |
| Missing | 499 (15.5%) | 22 (8.0%) | - |  |  |  |
| **Satisfaction with sex life, n (%)** |  |  |  |  |  |  |
| Happy | 2422 (75.4%) | 144 (52.4%) | 1 |  | 1 |  |
| Unhappy | 215 (6.7%) | 59 (21.5%) | 5.0 (3.5–7.3) | <0.001 | 3.5 (2.3–5.4) | <0.001 |
| Missing | 577 (18.0%) | 72 (26.2%) | - |  |  |  |
| **Type of PrEP use, n (%)** |  |  |  |  |  |  |
| Daily | 2279 (70.9%) | 161 (58.5%) | 1 |  | 1 |  |
| On demand / intermittent | 929 (28.9%) | 113 (41.1%) | 1.9 (1.4–2.6) | 0.001 | 1.4 (1.0–2.0) | 0.064 |
| Missing | 6 (0.2%) | 1 (0.4%) | - |  |  |  |
| **Number of anal sex partners within the last 6 months, n (%)** | | | | | | |
| 0–3 | 414 (12.9%) | 71 (25.8%) | 4.2 (2.8–6.3) | <0.001 | 1.7 (1.1–2.7) | 0.026 |
| 4–10 | 982 (30.6%) | 100 (36.4%) | 1.7 (1.2–2.5) | 0.004 | 1.1 (0.7–1.7) | 0.633 |
| >10 | 1699 (52.9%) | 97 (35.3%) | 1 |  | 1 |  |
| Missing | 119 (3.7%) | 7 (2.5%) | - |  |  |  |
| **Condom use while taking PrEP/since stopping PrEP, n (%)** | | | | | | |
| In about half the  times/sometimes/never | 2505 (77.9%) | 93 (33.8%) | 9.0 (6.4–12.7) | <0.001 | 7.6 (5.3–10.8) | <0.001 |
| Always/Often | 580 (18.0%) | 161 (58.5%) | 1 |  | 1 |  |
| Missing | 129 (4.0%) | 21 (7.6%) | - |  |  |  |
| **Recruited through, n (%) [multiple responses possible]** | | | | | | |
| Dating Apps | 2343 (72.9%) | 244 (88.7%) | - |  |  |  |
| Community Website | 203 (6.3%) | 7 (2.5%) | - |  |  |  |
| Anonymous Checkpoint | 69 (2.1%) | 5 (1.8%) | - |  |  |  |
| Friends | 309 (9.6%) | 18 (6.5%) | - |  |  |  |
| Missing | 427 (13.3%) | 13 (4.7%) | - |  |  |  |

^ꝉ^ Univariable logistic regression model, ^‡^ Multivariable logistic regression model adjusting for age, gender, country of origin, income, satisfaction with sex life, type of PrEP use, partner numbers and condom use. Two thousand and fifty-four current and 165 former PrEP users were included into the uni- and multivariable models. ^§^ Wald test. CI: confidence interval, IQR: interquartile range, OR: odds ratio, PrEP: pre-exposure prophylaxis

Appendix S5: Comparison of current PrEP and former PrEP users excluding participants from wave 2 if they had information missing as to whether they had participated in wave 1 (n = 2302)

|  | Current PrEP users, n (%) | Former PrEP users, n (%) | Univariable Analysis^ꝉ^ | | Multivariable Analysis^‡^ | |
| --- | --- | --- | --- | --- | --- | --- |
|  |  |  | OR (95% CI) | p-value^§^ | OR (95% CI) | p-value^§^ |
| **Total (n)** | 1979 | 323 |  |  |  |  |
| **Age (years)** |  |  |  |  |  |  |
| Median (IQR) | 37 (30–45) | 32 (26–41) |  |  |  |  |
| 18–29, n (%) | 435 (22.0%) | 124 (38.4%) | 1.8 (1.2–2.7) | 0.002 | 1.5 (1.0–2.4) | 0.057 |
| 30–39, n (%) | 736 (37.2%) | 103 (31.9%) | 1 |  | 1 |  |
| 40–49, n (%) | 532 (26.9%) | 67 (20.7%) | 1.1 (0.7–1.6) | 0.779 | 1.1 (0.7–1.8) | 0.602 |
| 50–80, n (%) | 276 (13.9%) | 29 (9.0%) | 0.9 (0.5–1.5) | 0.593 | 0.8 (0.4–1.5) | 0.438 |
| Missing, n (%) | - | - | - |  |  |  |
| **Gender, n (%)** |  |  |  |  |  |  |
| Male | 1942 (98.1%) | 320 (99.1%) | 1 |  | 1 |  |
| Female | - | - | - |  | - |  |
| Transgender / Non-Binary | 31 (1.6%) | 1 (0.3%) | 0.4 (0.1–3.2) | 0.410 | 0.3 (0.0–2.7) | 0.299 |
| Intersex | 5 (0.3%) | 2 (0.6%) | 7.7 (1.1–55.0) | 0.042 | 14.6 (1.7–122.6) | 0.014 |
| Missing | 1 (0.1%) | 0 (0.0%) | - |  |  |  |
| **Country of origin, n (%)** |  |  |  |  |  |  |
| Germany | 1355 (68.5%) | 204 (63.2%) | 1 |  | 1 |  |
| Outside Germany | 426 (21.5%) | 84 (26.0%) | 1.2 (0.9–1.8) | 0.226 | 0.9 (0.6–1.4) | 0.641 |
| Missing | 198 (10.0%) | 35 (10.8%) | - |  |  |  |
| **Annual gross income, n (%)** |  |  |  |  |  |  |
| <30,000 € | 583 (29.5%) | 125 (38.7%) | 1 |  | 1 |  |
| 30,000–49,000 € | 564 (28.5%) | 79 (24.5%) | 0.9 (0.6–1.3) | 0.471 | 1.3 (0.8–2.0) | 0.291 |
| ≥50,000 € | 763 (38.6%) | 97 (30.0%) | 0.9 (0.6–1.3) | 0.639 | 1.3 (0.8–2.0) | 0.321 |
| Missing | 69 (3.5%) | 22 (6.8%) | - |  |  |  |
| **Satisfaction with sex life, n (%)** |  |  |  |  |  |  |
| Happy | 1543 (78.0%) | 168 (52.0%) | 1 |  | 1 |  |
| Unhappy | 150 (7.6%) | 73 (22.6%) | 5.0 (3.5–7.2) | <0.001 | 3.8 (2.5–5.8) | <0.001 |
| Missing | 286 (14.5%) | 82 (25.4%) | - |  |  |  |
| **Duration of PrEP use, n (%)** |  |  |  |  |  |  |
| <3 months | 492 (24.9%) | 150 (46.4%) | 2.3 (1.7–3.1) | <0.001 | 1.4 (1.0–2.1) | 0.048 |
| ≥3 months | 1,487 (75.1%) | 173 (53.6%) | 1 |  | 1 |  |
| Missing | - | - | - |  |  |  |
| **Type of PrEP use, n (%)** |  |  |  |  |  |  |
| Daily | 1377 (69.6%) | 149 (46.1%) | 1 |  | 1 |  |
| On demand / intermittent | 598 (30.2%) | 170 (52.6%) | 2.7 (1.9–3.6) | <0.001 | 2.0 (1.4–2.9) | <0.001 |
| Missing | 4 (0.2%) | 4 (1.2%) | - |  |  |  |
| **Number of anal sex partners within the last 6 months, n (%)** | | | | | | |
| 0–3 | 283 (14.3%) | 100 (31.0%) | 4.5 (3.1–6.7) | <0.001 | 1.9 (1.2–3.0) | 0.005 |
| 4–10 | 707 (35.7%) | 118 (36.5%) | 1.7 (1.2–2.5) | 0.006 | 1.0 (0.7–1.5) | 0.982 |
| > 10 | 944 (47.7%) | 100 (31.0%) | 1 |  | 1 |  |
| Missing | 45 (2.3%) | 5 (1.5%) | - |  |  |  |
| **Condom use while taking PrEP / since stopping PrEP, n (%)** | | | | | | |
| In about half the  times/sometimes/never | 1528 (77.2%) | 97 (30.0%) | 1 |  | 6.9 (4.8–10.0) | <0.001 |
| Always / Often | 430 (21.7%) | 209 (64.7%) | 8.5 (6.0–11.9) | <0.001 | 1 |  |
| Missing | 21 (1.1%) | 17 (5.3%) | - |  |  |  |
| **Recruited through, n (%) [multiple responses possible]** | | | | | | |
| Dating Apps | 1738 (87.8% ) | 297 (92.0%) | - |  |  |  |
| Community Website | 76 (3.8%) | 5 (1.5%) | - |  |  |  |
| Anonymous Checkpoint | 41 (2.1%) | 3 (0.9%) | - |  |  |  |
| Friends | 190 (9.6%) | 22 (6.8%) | - |  |  |  |
| Missing | 29 (1.5%) | 6 (1.9%) | - |  |  |  |

^ꝉ^ Univariable logistic regression model, ^‡^ Multivariable logistic regression model adjusting for age, gender, country of origin, income, satisfaction with sex life, type of PrEP use, partner numbers and condom use. One thousand four hundred and thirty current and 186 former PrEP users were included into the uni- and multivariable models. ^§^ Wald test. CI: confidence interval, IQR: interquartile range, OR: odds ratio, PrEP: pre-exposure prophylaxis

Appendix S6: Reasons for stopping PrEP in former PrEP users stratified by duration of PrEP use

Former PrEP users who used PrEP for <3 months, multiple answers allowed

| **Reasons for stopping PrEP** | **Participants, n = 250** |
| --- | --- |
| **Reduced need for PrEP** | **135 (54.0%)** |
| Partner situation changed | 78 (31.2%) |
| Other prevention strategies are sufficient | 76 (30.4%) |
| **Logistic reasons** | **81 (32.4%)** |
| Difficulties obtaining PrEP^ꝉ^ | 30 (12.0%) |
| PrEP is unaffordable | 71 (28.4%) |
| **Negative attitudes towards PrEP** | **23 (9.2%)** |
| Afraid of stigma for taking PrEP | 6 (2.4%) |
| Thinking taking PrEP is immoral | 17 (6.8%) |
| **Reservations against characteristics of PrEP** | **130 (52.0%)** |
| Fear of long-term side effects | 80 (32.0%) |
| Not wanting to take a daily pill | 66 (26.4%) |
| Not wanting to take a chemical substance | 83 (33.2%) |
| **Experiencing side effects** | **50 (20.0%)** |
| **Biological reasons** | **30 (12.0%)** |
| Contracted too many STIs | 25 (10.0%) |
| Positive HIV test | 6 (2.4%) |
| **Other Reasons** | **16 (6.4%)** |
| **Missing** | **0 (0%)** |

^ꝉ^ The item ‘I have problems finding a doctor willing to prescribe PrEP’ included in this category was only available in wave 2 of the study. PrEP: pre-exposure prophylaxis, STI: sexually transmitted infection

Former PrEP users who used PrEP for ≥3 months, multiple answers allowed

| **Reasons for stopping PrEP** | **Participants, n = 275** |
| --- | --- |
| **Reduced need for PrEP** | **156 (56.7%)** |
| Partner situation changed | 111 (40.4%) |
| Other prevention strategies are sufficient | 70 (25.5%) |
| **Logistic reasons** | **108 (39.3%)** |
| Difficulties obtaining PrEP^ꝉ^ | 41 (14.9%) |
| PrEP is unaffordable | 86 (31.3%) |
| **Negative attitudes towards PrEP** | **25 (9.1%)** |
| Afraid of stigma for taking PrEP | 8 (2.9%) |
| Thinking taking PrEP is immoral | 19 (6.9%) |
| **Reservations against characteristics of PrEP** | **125 (45.5%)** |
| Fear of long-term side effects | 62 (22.5%) |
| Not wanting to take a daily pill | 75 (27.3%) |
| Not wanting to take a chemical substance | 66 (24.0%) |
| **Experiencing side effects** | **56 (20.4%)** |
| **Biological reasons** | **56 (20.4%)** |
| Contracted too many STIs | 42 (15.3%) |
| Positive HIV test | 15 (5.5%) |
| **Other Reasons** | **20 (7.3%)** |
| **Missing** | **0 (0%)** |

^ꝉ^ The item ‘I have problems finding a doctor willing to prescribe PrEP’ included in this category was only available in wave 2 of the study. PrEP: pre-exposure prophylaxis, STI: sexually transmitted infection

Appendix S7: Reasons for stopping PrEP in former PrEP users stratified by type of PrEP use

Former daily PrEP users, multiple answers allowed

| **Reasons for stopping PrEP** | **Participants, n = 245** |
| --- | --- |
| **Reduced need for PrEP** | **126 (51.4%)** |
| Partner situation changed | 88 (35.9%) |
| Other prevention strategies are sufficient | 55 (22.4%) |
| **Logistic reasons** | **90 (36.7%)** |
| Difficulties obtaining PrEP^ꝉ^ | 29 (11.8%) |
| PrEP is unaffordable | 75 (30.6%) |
| **Negative attitudes towards PrEP** | **20 (8.2%)** |
| Afraid of stigma for taking PrEP | 4 (1.6%) |
| Thinking taking PrEP is immoral | 16 (6.5%) |
| **Reservations against characteristics of PrEP** | **109 (44.5%)** |
| Fear of long-term side effects | 70 (28.6%) |
| Not wanting to take a daily pill | 59 (24.1%) |
| Not wanting to take a chemical substance | 61 (24.9%) |
| **Experiencing side effects** | **68 (27.8%)** |
| **Biological reasons** | **48 (19.6%)** |
| Contracted too many STIs | 39 (15.9%) |
| Positive HIV test | 9 (3.7%) |
| **Other Reasons** | **25 (10.2%)** |
| **Missing** | **0 (0%)** |

^ꝉ^ The item ‘I have problems finding a doctor willing to prescribe PrEP’ included in this category was only available in wave 2 of the study. PrEP: pre-exposure prophylaxis, STI: sexually transmitted infection

Former demand/intermittent PrEP users, multiple answers allowed

| **Reasons for stopping PrEP** | **Participants, n = 275** |
| --- | --- |
| **Reduced need for PrEP** | **162 (58.9%)** |
| Partner situation changed | 100 (36.4%) |
| Other prevention strategies are sufficient | 89 (32.4%) |
| **Logistic reasons** | **96 (34.9%)** |
| Difficulties obtaining PrEP^ꝉ^ | 39 (14.2%) |
| PrEP is unaffordable | 80 (29.1%) |
| **Negative attitudes towards PrEP** | **26 (9.5%)** |
| Afraid of stigma for taking PrEP | 9 (3.3%) |
| Thinking taking PrEP is immoral | 19 (6.9%) |
| **Reservations against characteristics of PrEP** | **145 (52.7%)** |
| Fear of long-term side effects | 72 (26.2%) |
| Not wanting to take a daily pill | 82 (29.8%) |
| Not wanting to take a chemical substance | 85 (30.9%) |
| **Experiencing side effects** | **35 (12.7%)** |
| **Biological reasons** | **37 (13.5%)** |
| Contracted too many STIs | 27 (9.8%) |
| Positive HIV test | 12 (4.4%) |
| **Other Reasons** | **10 (3.6%)** |
| **Missing** | **0 (0%)** |

^ꝉ^ The item ‘I have problems finding a doctor willing to prescribe PrEP’ included in this category was only available in wave 2 of the study. PrEP: pre-exposure prophylaxis, STI: sexually transmitted infection

Appendix S8: Reasons for stopping PrEP in former PrEP users stratified by sexual behaviour

Former PrEP users with inconsistent condom use and ≥4 anal/vaginal sex partners within the last 6 months

| **Reasons for stopping PrEP** | **Participants, n = 114** |
| --- | --- |
| **Reduced need for PrEP** | **44 (38.6%)** |
| Partner situation changed | 40 (35.1%) |
| Other prevention strategies are sufficient | 11 (9.6%) |
| **Logistic reasons** | **53 (46.5%)** |
| Difficulties obtaining PrEP^ꝉ^ | 26 (22.8%) |
| PrEP is unaffordable | 38 (33.3%) |
| **Negative attitudes towards PrEP** | **12 (10.5%)** |
| Afraid of stigma for taking PrEP | 7 (6.1%) |
| Thinking taking PrEP is immoral | 7 (6.1%) |
| **Reservations against characteristics of PrEP** | **53 (46.5%)** |
| Fear of long-term side effects | 28 (24.6%) |
| Do not feel adequately protected by PrEP | 9 (7.9%) |
| Not wanting to take a daily pill | 33 (28.9%) |
| Not wanting to take a chemical substance | 28 (24.6%) |
| **Experiencing side effects** | **23 (20.2%)** |
| **Biological reasons** | **16 (14.0%)** |
| Contracted too many STIs | 16 (14.0%) |
| Positive HIV test | 0 (0%) |
| **Other Reasons** | **7 (6.1%)** |
| **Missing** | **0 (0%)** |

^ꝉ^ The item ‘I have problems finding a doctor willing to prescribe PrEP’ included in this category was only available in wave 2 of the study. PrEP: pre-exposure prophylaxis, STI: sexually transmitted infection

Former PrEP users with consistent condom use and/or ≤3 anal/vaginal sex partners within the last 6 months

| **Reasons for stopping PrEP** | **Participants, n = 495** |
| --- | --- |
| **Reduced need for PrEP** | **255 (51.5%)** |
| Partner situation changed | 153 (30.9%) |
| Other prevention strategies are sufficient | 139 (28.1%) |
| **Logistic reasons** | **138 (27.9%)** |
| Difficulties obtaining PrEP^ꝉ^ | 46 (9.3%) |
| PrEP is unaffordable | 121 (24.4%) |
| **Negative attitudes towards PrEP** | **36 (7.3%)** |
| Afraid of stigma for taking PrEP | 7 (1.4%) |
| Thinking taking PrEP is immoral | 29 (5.9%) |
| **Reservations against characteristics of PrEP** | **209 (42.2%)** |
| Fear of long-term side effects | 119 (24.0%) |
| Do not feel adequately protected by PrEP | 41 (8.3%) |
| Not wanting to take a daily pill | 109 (22.0%) |
| Not wanting to take a chemical substance | 122 (24.6%) |
| **Experiencing side effects** | **83 (16.8%)** |
| **Biological reasons** | **70 (14.1%)** |
| Contracted too many STIs | 51 (10.3%) |
| Positive HIV test | 21 (4.2%) |
| **Other Reasons** | **29 (5.9%)** |
| **Missing** | **69 (13.9%)** |

^ꝉ^ The item ‘I have problems finding a doctor willing to prescribe PrEP’ included in this category was only available in wave 2 of the study. PrEP: pre-exposure prophylaxis, STI: sexually transmitted infection

Appendix S 9: Participants in wave 2 who reported information on possible side effects (including those who indicated participating in wave 1)

|  | All participants in wave 2 who reported information on possible side effects, n (%) |
| --- | --- |
|  |  |
| **Total (n)** | 2675 |
| **Age (years)** |  |
| Median (IQR) | 37 (30–45) |
| 18–29, n (%) | 602 (22.5%) |
| 30–39, n (%) | 966 (36.1%) |
| 40–49, n (%) | 735 (27.5%) |
| 50–80, n (%) | 372 (13.9%) |
| Missing | 0 (0%) |
| **Gender, n (%)** |  |
| Male | 2291 (85.65%) |
| Female | 0 (0%) |
| Transgender/Non-Binary | 35 (1.3%) |
| Intersex | 7 (0.3%) |
| Missing | 342 (12.8%) |
| **Country of origin, n (%)** |  |
| Germany | 1797 (67.2%) |
| Outside Germany | 566 (21.2%) |
| Missing | 312 (11.7%) |
| **Annual gross income, n (%)** |  |
| <30,000 € | 783 (29.3%) |
| 30,000–49,000 € | 729 (27.3%) |
| ≥50,000 € | 1,004 (37.5%) |
| Missing | 159 (5.9%) |
| **Satisfaction with sex life, n (%)** |  |
| Happy | 2008 (75.1%) |
| Unhappy | 262 (9.8%) |
| Missing | 405 (15.1%) |
| **Type of PrEP use, n (%)** |  |
| Daily | 1819 (68.0%) |
| On demand/intermittent | 845 (31.6%) |
| Missing | 11 (0.4%) |
| **Number of anal sex partners within the last 6 months, n (%)** |  |
| 0–3 | 432 (16.1%) |
| 4–10 | 922 (34.5%) |
| >10 | 1151 (47.2%) |
| Missing | 59 (2.2%) |
| **Condom use while taking PrEP / since stopping PrEP, n (%)** |  |
| In about half the  times/sometimes/never | 1943 (72.6%) |
| Always/Often | 688 (25.7%) |
| Missing | 44 (1.6%) |
| **Recruited through, n (%) [multiple responses possible]** |  |
| Dating Apps | 2293 (85.7%) |
| Community Website | 113 (4.2%) |
| Anonymous Checkpoint | 53 (2.0%) |
| Friends | 239 (8.9%) |
| Missing | 98 (3.7%) |

Appendix S 10: Comparison of participants, who are in- and excluded in the logistic regression analysis

|  | Excluded participants, n (%)^1^ | Included participants, n (%) |
| --- | --- | --- |
| **Total (n)** | 2499 | 2958 |
| **Age (years)** |  |  |
| Median (IQR) | 36 (29-44) | 37 (30-45) |
| 18–29, n (%) | 526 (26.7%) | 650 (22.0%) |
| 30–39, n (%) | 719 (36.5%) | 1077 (36.4%) |
| 40–49, n (%) | 468 (23.7%) | 840 (28.4%) |
| 50–80, n (%) | 259 (13.1%) | 391 (13.2%) |
| Missing, n (%) | 527 | - |
| **Gender, n (%)** |  |  |
| Male | 1907 (97.7%) | 2929 (99.0%) |
| Female | 2 (0.1%) | 0 (0%) |
| Transgender / Non-Binary | 32 (1.6%) | 24 (0.8%) |
| Intersex | 10 (0.5%) | 5 (0.2%) |
| Missing | 548 | - |
| **Country of origin, n (%)** |  |  |
| Germany | 588 (70.8%) | 2268 (76.7%) |
| Outside Germany | 242 (29.2%) | 690 (23.3%) |
| Missing | 1669 | - |
| **Annual gross income, n (%)** |  |  |
| <30,000 € | 385 (36.3%) | 801 (27.1%) |
| 30,000–49,000 € | 326 (30.7%) | 910 (30.8%) |
| ≥50,000 € | 351 (33.1%) | 1247 (42.2%) |
| Missing | 1437 | - |
| **Satisfaction with sex life, n (%)** |  |  |
| Happy | 758 (87.6%) | 2656 (89.8%) |
| Unhappy | 107 (12.4%) | 302 (10.2%) |
| Missing | 1634 | - |
| **Duration of PrEP use, n (%)** |  |  |
| <3 months | 531 (29.5%) | 739 (25.0%) |
| ≥3 months | 1270 (70.5%) | 2219 (75.0%) |
| Missing | 698 | - |
| **Type of PrEP use, n (%)** |  |  |
| Daily | 1130 (62.2%) | 2067 (69.9%) |
| On demand/intermittent | 687 (37.8%) | 891 (30.1%) |
| Missing | 682 | - |
| **Number of anal sex partners within the previous 6 months, n (%)** |  |  |
| 0–3 | 354 (21.9%) | 435 (14.7%) |
| 4–10 | 579 (35.8%) | 957 (32.4%) |
| > 10 | 684 (42.3%) | 1566 (52.9%) |
| Missing | 882 | - |
| **Condom use while taking PrEP / since stopping PrEP, n (%)** |  |  |
| In about half the  time/sometimes/never | 1124 (71.7%) | 2200 (74.4%) |
| Always/Often | 444 (28.3%) | 758 (25.6%) |
| Missing | 931 | - |

^1^ To allow comparison of the variable distribution between included and excluded participants, patients with missing values were not included in the calculation of the percentage values.

Appendix S 11: Sensitivity analysis of multivariable logistic regression model including missing values as a separate category

|  | Current PrEP users, n (%) | Former PrEP users, n (%) | Univariable Analysis^ꝉ^ | | Multivariable Analysis^‡^ | |
| --- | --- | --- | --- | --- | --- | --- |
|  |  |  | OR (95% CI) | p-value^§^ | OR (95% CI) | p-value^§^ |
| **Total (n)** | 4848 | 609 |  |  |  |  |
| **Age (years)** |  |  |  |  |  |  |
| Median (IQR) | 37 (30–45) | 33 (27–41) |  |  |  |  |
| 18–29, n (%) | 967 (19.9%) | 209 (34.3%) | 2.0 (1.6–2.5) | <0.001 | 1.7 (1.3–2.2) | <0.001 |
| 30–39, n (%) | 1620 (33.4%) | 176 (28.9%) | 1 |  | 1 |  |
| 40–49, n (%) | 1199 (24.7%) | 109 (17.9%) | 0.8 (0.7–1.1) | 0.163 | 0.9 (0.7–1.2) | 0.631 |
| 50–80, n (%) | 597 (12.3%) | 53 (8.7%) | 0.8 (0.6–1.1) | 0.218 | 0.9 (0.6–1.3) | 0.617 |
| Missing, n (%) | 465 (9.6%) | 62 (10.2%) | 1.2 (0.9 – 1.7) | 0.191 | 1.0 (0.3 – 3.4) | 0.954 |
| **Gender, n (%)** |  |  |  |  |  |  |
| Male | 4299 (88.7%) | 537 (88.2%) | 1 |  | 1 |  |
| Female | 2 (0.0%) | 0 (0.0%) | - |  |  |  |
| Transgender / Non-Binary | 52 (1.1%) | 4 (0.7%) | 0.6 (0.2–1.7) | 0.352 | 0.5 (0.2–1.4) | 0.194 |
| Intersex | 12 (0.2%) | 3 (0.5%) | 2.0 (0.6–7.1) | 0.284 | 2.1 (0.6–8.0) | 0.272 |
| Missing | 483 (10.0%) | 65 (10.7%) | 1.1 (0.8 – 1.4) | 0.594 | 1.1 (0.3 – 4.1) | 0.858 |
| **Country of origin, n (%)** |  |  |  |  |  |  |
| Germany | 2531 (52.2%) | 325 (53.4%) | 1 |  | 1 |  |
| Outside Germany | 813 (16.8%) | 119 (19.5%) | 1.1 (0.9–1.4) | 0.253 | 0.8 (0.6–1.0) | 0.102 |
| Missing | 1504 (31.0%) | 165 (27.1%) | 0.9 (0.7 – 1.0) | 0.119 | 0.7 (0.5 – 0.9) | 0.022 |
| **Annual gross income, n (%)** |  |  |  |  |  |  |
| <30,000 € | 1005 (20.7%) | 181 (29.7%) | 1 |  | 1 |  |
| 30,000–49,000 € | 1104 (22.8%) | 132 (21.7%) | 0.7 (0.5–0.8) | 0.001 | 1.0 (0.7–1.3) | 0.798 |
| ≥50,000 € | 1441 (29.7%) | 157 (25.8%) | 0.6 (0.5–0.8) | <0.001 | 0.9 (0.7–1.2) | 0.501 |
| Missing | 1298 (26.8%) | 139 (22.8%) | 0.6 (0.5 – 0.8) | <0.001 | 0.4 (0.3 – 0.6) | <0.001 |
| **Satisfaction with sex life, n (%)** |  |  |  |  |  |  |
| Happy | 3144 (64.9%) | 270 (44.3%) | 1 |  | 1 |  |
| Unhappy | 298 (6.1%) | 111 (18.2%) | 4.3 (3.4–5.6) | <0.001 | 3.1 (2.3–4.1) | <0.001 |
| Missing | 1406 (29.0%) | 228 (37.4%) | 1.9 (1.6 – 2.3) | <0.001 | 1.9 (1.4 – 2.4) | <0.001 |
| **Duration of PrEP use, n (%)** |  |  |  |  |  |  |
| <3 months | 1020 (21.0%) | 250 (41.1%) | 2.9 (2.4–3.4) | <0.001 | 1.9 (1.6–2.4) | 0.001 |
| ≥3 months | 3214 (66.3%) | 275 (45.2%) | 1 |  | 1 |  |
| Missing | 614 (12.7%) | 84 (13.8%) | 1.6 (1.2 – 2.1) | <0.001 | 0.5 (0.1 – 1.6) | 0.225 |
| **Type of PrEP use, n (%)** |  |  |  |  |  |  |
| Daily | 2952 (60.9%) | 245 (40.2%) | 1 |  | 1 |  |
| On demand/intermittent | 1302 (26.9%) | 276 (45.3%) | 2.6 (2.1–3.1) | <0.001 | 2.0 (1.7–2.5) | <0.001 |
| Missing | 594 (12.3%) | 88 (14.4%) | 1.8 (1.4 – 2.3) | <0.001 | 4.5 (1.6 – 12.8) | 0.004 |
| **Number of anal sex partners within the previous 6 months, n (%)** |  |  |  |  |  |  |
| 0–3 | 622 (12.8%) | 167 (27.4%) | 3.5 (2.8–4.4) | <0.001 | 1.5 (1.1–1.9) | 0.006 |
| 4–10 | 1355 (27.9%) | 181 (29.7%) | 1.7 (1.4–2.2) | <0.001 | 1.1 (0.8–1.3) | 0.701 |
| > 10 | 2089 (43.1%) | 161 (26.4%) | 1 |  | 1 |  |
| Missing | 782 (16.1%) | 100 (16.4%) | 1.7 (1.3 – 2.2) | <0.001 | 0.9 (0.5 – 1.7) | 0.678 |
| **Condom use while taking PrEP / since stopping PrEP, n (%)** |  |  |  |  |  |  |
| In about half the  time/sometimes/never | 3164 (65.3%) | 160 (26.3%) | 1 |  | 1 |  |
| Always/Often | 874 (18.0%) | 328 (53.9%) | 7.4 (6.1–9.1) | <0.001 | 5.9 (4.7–7.3) | <0.001 |
| Missing | 810 (16.7%) | 121 (19.9%) | 3.0 (2.3 – 3.8) | <0.001 | 5.3 (3.2 – 8.8) | <0.001 |

^ꝉ^ Univariable logistic regression model, ^‡^ Multivariable logistic regression model adjusting for age, gender, country of origin, income, satisfaction with sex life, type of PrEP use, partner numbers and condom use. Four thousand eight hundred and fourty-eight current and 609 former PrEP users were included into the uni- and multivariable models. ^§^ Wald test. CI: confidence interval, IQR: interquartile range, OR: odds ratio, PrEP: pre-exposure prophylaxis
